# New South London Hospital for Women: The Operation Unit

**Published:** 1916-07-15

**Authors:** M. Collins

**Affiliations:** 61 Old Broad Street, London, E.C.


### THE EDITOR'S LETTER-BOX.

### New South London Hospital for Women: the Operation Unit.

*To the Editor of* The Hospital.

Sir,—I have perused with great interest your article on the South London Hospital for Women published on pages 349 and 350 of your issue under date the 8th inst., and I note your somewhat adverse criticism of the operating block. I should like to afford you some further information with reference to this particular point.

All the matters referred to in your criticism were fully discussed by the lady surgeons, and after mature consideration the operating block was carried out as now installed. The arrangement was as follows : The surgeon enters the operating block and at once goes to the retiring room which is fitted up with lavatory, hot and cold water, towel heater, etc., and here changes such part of her apparel as may be necessary, puts on overalls, sleeves, etc., and in every way prepares herself before entering the operating room. The main steriliser is in a room exactly opposite this small retiring room, and here all the overalls, sleeves, and other parts of the surgeon's over-clothing is sterilised and ready for use when required. The lavatories and sinks which are in a small annexe, and practically form a component part of the large operating room, are merely for the use of nurses and surgeons after operations. The small annexe at the entrance to the large theatre which is marked "steriliser" on the plan is merely for small sterilisers for instruments, gloves, etc., and practically speaking perhaps I ought not to have worded this as I have. Now with reference to the anæsthetic room, it was deemed that this need only be used for this specific purpose when the two operating rooms are simultaneously in use, which will very seldom, indeed hardly ever, perhaps happen. In all other cases the minor theatre can be used for the anæsthetist, thereby avoiding passing back through the anæsthetic-room after an operation. With regard to a bathroom, this question was much debated, and the ladies deemed that, as this was entirely a women's hospital, and as there would be no mixture of the sexes within its walls after it is once opened, if required they could well make use of the bathroom which you will note is placed immediately next to the theatre block. The arrangement as carried out and sundry other arrangements of the building have been the results not of the lack of knowledge but of much consideration on the part of the lady surgeons and physicians, and a desire to economise as much as possible.

Permit me to thank you for the great trouble you have taken in this matter, but to express regret that you did not afford me an opportunity of seeing your "proof" before it was issued.—I am, Sir, yours truly,

M. Collins.

61 Old Broad Street, London, E.C.,
July 11, 1916.

[We are obliged to Mr. Collins for the full explanation contained in his letter. We are afraid, however, experts will agree that the explanation enforces the criticism and caution we ventured to give in describing the plans. We are convinced that the consultants referred to at the conferences in question brought much knowledge to bear on their deliberations, but we fear that in practice economy may have blanketed out knowledge where important details had to be settled. It is not practicable to send proofs as suggested.—Ed. The Hospital.]

